# Preclinical Efficacy of Pro- and Anti-Angiogenic Peptide Hydrogels to Treat Age-Related Macular Degeneration

**DOI:** 10.3390/bioengineering8120190

**Published:** 2021-11-23

**Authors:** Amanda Acevedo-Jake, Siyu Shi, Zain Siddiqui, Sreya Sanyal, Rebecca Schur, Simon Kaja, Alex Yuan, Vivek A. Kumar

**Affiliations:** 1 Department of Biomedical Engineering, New Jersey Institute of Technology, Newark, NJ 07102, USA; mauvocado@gmail.com (A.A.-J.); zs67@njit.edu (Z.S.); 2 Stanford School of Medicine, Stanford University, Stanford, CA 94305, USA; siyushi@stanford.edu; 3 Department of Biology, New Jersey Institute of Technology, Newark, NJ 07102, USA; ss3742@njit.edu; 4 Cole Eye Institute, Cleveland Clinic Lerner College of Medicine, Cleveland, OH 44195, USA; SCHURR@ccf.org (R.S.); yuana@ccf.org (A.Y.); 5 Research & Development Division, Experimentica Ltd., 70211 Kuopio, Finland; kaja@experimentica.com; 6 Department of Ophthalmology, Loyola University Chicago, Maywood, IL 60153, USA; 7 Department of Chemical Engineering, New Jersey Institute of Technology, Newark, NJ 07102, USA; 8 Department of Restorative Dentistry, Rutgers School of Dental Medicine, Newark, NJ 07102, USA

**Keywords:** wet age-related macular degeneration, pro-angiogenic, anti-angiogenic, hydrogel, biomaterials, tissue regeneration, multi-functional scaffolds

## Abstract

Pro-angiogenic and anti-angiogenic peptide hydrogels were evaluated against the standard of care wet age-related macular degeneration (AMD) therapy, Aflibercept (Eylea^®^). AMD was modeled in rats (laser-induced choroidal neovascularization (CNV) model), where the contralateral eye served as the control. After administration of therapeutics, vasculature was monitored for 14 days to evaluate leakiness. Rats were treated with either a low or high concentration of anti-angiogenic peptide hydrogel (0.02 wt% 8 rats, 0.2 wt% 6 rats), or a pro-angiogenic peptide hydrogel (1.0 wt% 7 rats). As controls, six rats were treated with commercially available Aflibercept and six with sucrose solution (vehicle control). Post lasering, efficacy was determined over 14 days via fluorescein angiography (FA) and spectral-domain optical coherence tomography (SD-OCT). Before and after treatment, the average areas of vascular leak per lesion were evaluated as well as the overall vessel leakiness. Unexpectedly, treatment with pro-angiogenic peptide hydrogel showed significant, immediate improvement in reducing vascular leak; in the short term, the pro-angiogenic peptide performed better than anti-angiogenic peptide hydrogel and was comparable to Aflibercept. After 14 days, both the pro-angiogenic and anti-angiogenic peptide hydrogels show a trend of improvement, comparable to Aflibercept. Based on our results, both anti-angiogenic and pro-angiogenic peptide hydrogels may prove good therapeutics in the future to treat wet AMD over a longer-term treatment period.

## 1. Introduction

Age-related macular degeneration (AMD) affects 11 million patients in the United States and is the leading cause of visual disability in industrialized countries [1]. A subtype of AMD, wet AMD (neovascular AMD), is characterized by abnormal growth of vessels in choroidal and retinal circulations promoted by vascular endothelial growth factors (VEGF) [2]. Neovascularization coupled with high vascular permeability leads to macular edema or hemorrhage and eventual visual impairment [3].

Anti-VEGF is the most common treatment paradigm for wet AMD and many other retinal diseases, including proliferative diabetic retinopathy and retinal vein occlusions [4]. Current anti-VEGF therapies require regular intravitreal injections that are associated with sight-threatening complications including endophthalmitis [5]. Administration routes alternative to intravitreal injections such as topical administrations have been unsuccessful to date due to the difficulty in reaching choroidal blood vessels [6]. Recently, a new promising strategy has emerged capitalizing on the sustained release of anti-VEGF agents to the posterior segment of the eye, employing implants, nano-formulations, and hydrogels [6]. While the results of research in the area are encouraging [6], wet AMD therapies are still faced with significant challenges in their inherent bioactivity, payload quantity, targeted delivery, sustained release, biocompatibility, and optical clarity; therapeutics which successfully address these shortcomings will have improved performance and will result in better long-term patient outcomes.

**Figure 1:**
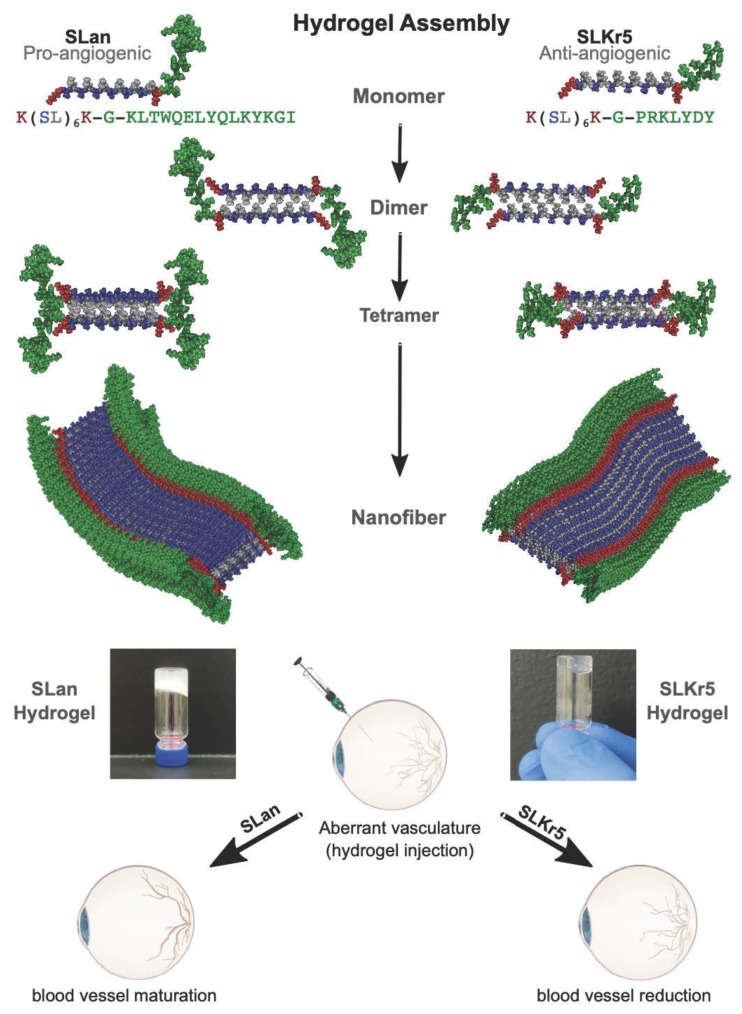
Schematic of the proposed assembly mechanism of the hydrogels.

Peptide-based hydrogels successfully address many of the challenges above, and additionally are readily synthesized at low-cost and high purity, can be conjugated to other polymers, fluorophores or chemical moieties in a straightforward manner to alter or improve their physical properties, can be modified to be persistent, biodegradable or responsive to stimuli, and can serve as excellent biomimetic scaffolds—all characteristics which prime their use as biomaterials for medical applications [7,8,9,10,11,12,13,14]. While many strategies developing covalently crosslinked hydrogels have shown great potential in the biomedical field [15,16,17,18,19,20], non-covalently crosslinked hydrogels, whose assembly and structure are solely guided by weak supramolecular interactions, confer additional advantages such as self-healing, high elasticity, and shear thinning [10,17,21]. Rational design of the base peptide sequence governs both the final structure and the inherent functionality of the final material, while the ability to easily tune peptide sequences facilitates modularity in these platforms [10,22,23,24,25]. Multi-domain peptide MDP hydrogels exemplify many of the characteristics above and are composed of an alternating A-B-A sequence motif, where the terminal A blocks contain either positively or negatively charged amino acids (ex. K/R or E/D), and the B midblock domain contains a repetitive beta-sheet domain of alternating hydrophobic and hydrophilic amino acids (ex. (SL)_6_) [23,24,26,27,28]. Upon aqueous dissolution, beta-sheet monomers spontaneously associate into dimers to exclude solvent and form a hydrophobic core (Figure 1) while hydrophilic residues associate with solvent. Further assembly of dimers produces short fibers, though significant anisotropic fiber extension is prevented by the proximity of many charged A domains [29,30,31]. Subsequent addition of charged ions, drugs or other polymers facilitates terminal charge shielding in these domains and allows for unidirectional fiber extension as well as three-dimensional intertangling of multiple fibers, ultimately giving rise to stiff, optically clear, thixotropic hydrogels [23,27,32,33,34,35,36]. While this base sequence has shown excellent promise as a scaffold for wound healing, drug delivery and tissue regeneration [24,26,27,28,32,35,36,37,38], additional functionality can be incorporated by appending short epitopes at one of the termini, generating new A-B-A-C-type hydrogels which can be used to promote neurogenesis or dentinogenesis, modulate inflammation, reduce bacterial load or regulate lipoprotein homeostasis [39,40,41,42,43,44]. Of these designer hydrogels, versions which are either pro- or anti-angiogenic have recently shown great potential for tissue regeneration in several disparate biomedical applications [40,43,45,46,47,48,49,50].

In this study, we investigated the preclinical efficacy of two novel peptide hydrogels, one containing a pro-angiogenic [43] motif and the other an anti-angiogenic motif in a rat wet AMD model (Figure 1) [6]. Both novel peptide hydrogels demonstrate biocompatibility, targeted delivery, sustained release, and optical clarity—important design criteria for biomaterials for wet AMD treatment. Surprisingly, the overall efficacy of the pro-angiogenic peptide hydrogel was significantly improved compared to the anti-angiogenic peptide hydrogel, and its performance was found comparable to Aflibercept (Eylea^®^), an approved treatment for wet AMD. Our results suggest a possible new protective role of VEGF in stabilizing neovascularization and reducing vascular permeability in wet AMD.

## 2. Materials and Methods

### 2.1. Peptide Preparation and Characterization

Pro-angiogenic peptide SLan (peptide sequence K(SL)_6_K–G–KLTWQELYQLKYKGI) and anti-angiogenic peptide SLKr5 (peptide sequence K(SL)_6_K–G–PRKLYDY) were synthesized with a CEM LibertyBlue solid phase peptide synthesizer using standard Fmoc chemistry. Peptide purity was verified >85% by an Agilent 1100 series HPLC instrument with an Agilent (Santa Clara, CA, USA) C3 reverse phase column, and products were monitored by UV at 280 nm. The identity of the peptides was verified with an Orbitrap Q Exactive LC/MS (Thermo Scientific, Waltham, MA, USA) instrument. Lyophilized pro-angiogenic and anti-angiogenic peptides were formulated as gels with the addition of 298 mM sucrose and passed through a 0.22 um filter for sterilization, and multivalent counterions (PO_4_^3−^) in 1X PBS (autoclaved), to maintain osmolarity, shield charges, and form salt-bridges between the terminal lysines of the peptides. Note: Sterile filtering should not affect the overall structure of the peptides or hydrogels, as the gel is formed through non-covalent interactions as it self-assembles. Chemical characterization of the pro-angiogenic peptide has been previously reported [43]. Optical transparency of gels was observed during optical imaging in the eye; optimization of imaging with trial eyes led to the choice of concentrations used, which were noted to be optically transparent both in vivo and in vitro. Two concentrations of anti-angiogenic peptide hydrogel (low concentration—0.02 wt% and high concentration—0.2 wt%) and one concentration of pro-angiogenic peptide hydrogel (1.0 wt%) were thereby selected to proceed with in vivo studies because these hydrogels remain optically clear at these concentrations.

### 2.2. Rat Laser-Induced Choroidal Neovascularization Model

A rat laser-induced CNV model is a well-established animal model for wet AMD. All animal studies were in accordance with State and Federal Guidelines and approved by the IACUC committee of Experimentica Ltd. (Forest Park, IL, USA). Briefly, animals were anesthetized, given a subcutaneous injection of ketamine and medetomidine, and a 0.5% solution of tropicamide (Alcon, Fort Worth, TX, USA) was applied to the cornea to dilate the pupil. Laser photocoagulation (power 150 mW, 100 uM spot size, 100 ms duration) was performed once using a 532 nm diode laser (Novus Spectra, Lumenis, Israel) attached to a slit lamp. A coverslip and Viscotears^®^ gel (Novartis, Cambridge, MA, USA) were used to applanate the cornea, four laser lesions were performed in each eye, and anesthesia immediately reversed. Immediately after lasering, unilateral intravitreal (IVT) administrations of 5 μL peptide hydrogels each were performed via a glass microsyringe (33 G needle, Hamilton Bonaduz AG, Bonaduz, Switzerland). Eight male rats were treated with low concentration anti-angiogenic peptide hydrogel (0.02 wt%), six with high concentration anti-angiogenic peptide hydrogel (0.2 wt%), seven with pro-angiogenic peptide hydrogel (1 wt%), six with Aflibercept (200 μg), and six with 298 mM sucrose solution (vehicle). The contralateral eye served as control. Rats were followed using in vivo imaging for 14 days via fluorescein angiography (FA) and spectral-domain optical coherence tomography (SD-OCT).

### 2.3. Analysis of Choroidal Neovascularization Lesions with In Vivo Imaging

To determine the preclinical efficacy of pro-angiogenic and anti-angiogenic hydrogels, the CNV lesions were monitored using spectral-domain optical coherence tomography (SD-OCT) and fluorescein angiography (FA) at baseline on day 0 (after lasering), and at follow-up days 3, day 7, and day 14 (end of the study period). Rats received subcutaneous injections of 1 mL of 5% fluorescein sodium salt (Sigma-Aldrich Finland Oy, cat. No. F6377), as is typical for rodents [51,52,53]. Vascular leakage was examined using a Heidelberg Spectralis HRA2 system (Heidelberg Engineering, Germany). CNV lesions and retinal thickness were monitored using an Envisu R2210 SD-OCT system (Bioptigen Inc./Leica Microsystems, Morrisville, NC, USA). For imaging, the rat was placed into the holder and the imaging systems aligned with the first infrared reflectance image taken from the system, then sodium fluorescein was administered and consecutive FA images taken every 60 s from the retinal and choroidal focus level for a period of 5 min from the sodium fluorescein injection. Qualitatively, lasered spots were evaluated based on the presence or absence of a retinal vascular leak. The presence of CNV was identified from lasered spots that have a leakage as observed by comparing the dynamics of the fluorescein signal in a series of fundus FA images. SD-OCT imaging was used as a secondary confirmation of CNV or in questionable FA, where the presence of intraretinal fluid in SD- OCT images would suggest the presence of CNV. Quantitatively, vascular leakage was measured using FIJI (v. 2.0) software by outlining the area of vascular leak manually from the last FA image of each imaging session. All researchers were blinded on the treatment each animal received during measurement.

### 2.4. Data Analysis

Quantitative data were plotted, analyzed, and presented as mean ± standard deviation (SD) or standard error of mean (SEM). Lesion size as well as leakiness was compared within each group and between each group, and accordingly, parametric data was analyzed using one-way ANOVA test, while non-parametric data was analyzed using Kruskal–Wallis ANOVA. Multiple comparison tests were performed as appropriate, and data with common Greek symbols showed no statistical significance at *p* < 0.05 level.

## 3. Results

### 3.1. Tolerability of Peptide Hydrogels

Therapeutic utility and tolerability were evaluated in the rat laser-induced choroidal neovascularization model, a commonly used animal model for wet AMD [54]. No adverse reactions due to the study compounds were observed. Animals were monitored for any notable changes in body weight and animal behavior, as well as signs of inflammation in the eye for the duration of the study period. Rat weight was not affected by the treatments (*p* = 0.542, Supplemental Figure S1), and no redness, swelling or loss of function was noted in any of the eyes, nor were any gross morphology changes noted. At the time of explant, gross observation of animal and organs revealed no untoward observation. We observed optical transparency of gels during optical imaging in the eye; optimization of imaging with trial eyes led to the choice of concentrations used, which were noted to be optically transparent both in vivo and in vitro.

### 3.2. Qualitative Analysis of CNV Lesions

**Figure 2:**
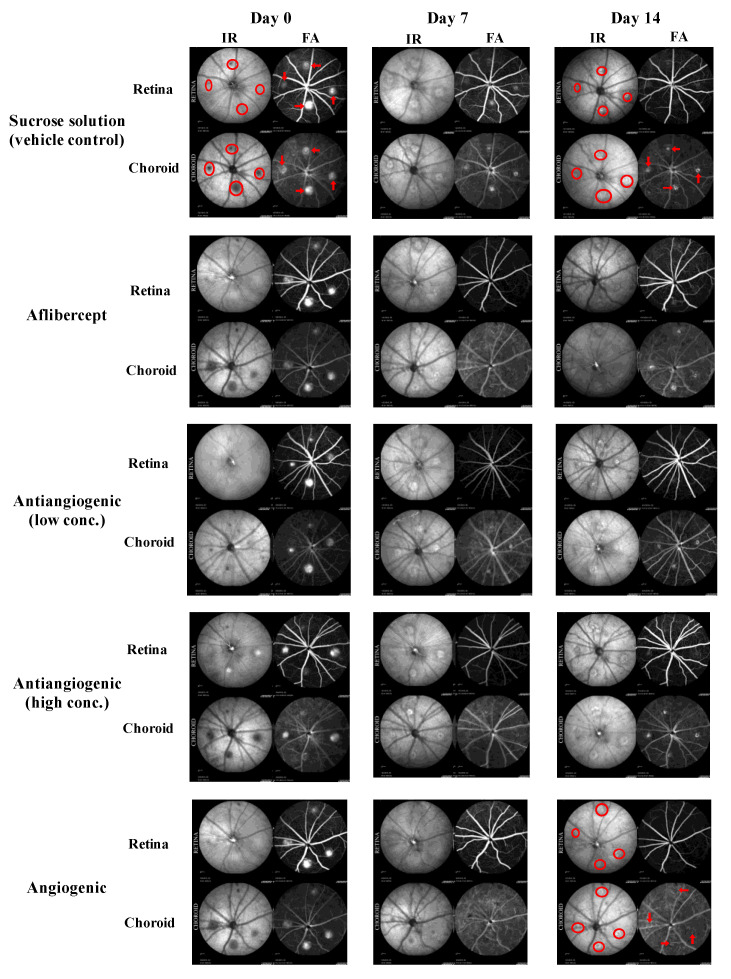
Representative infrared reflectance (IR) and fluorescein angiography (FA) images taken 5 min post sodium fluorescein injection for both the retinal and the choroidal focus planes in CNV induced eyes. Lasered spots in the choroidal and retinal focus planes are indicated by red circles at day 0 for reference and appear as a shadowed area by IR. Bright spots in these focus planes during FA imaging show areas of vascular leakage, which are indicated by red arrows in day 0. Day 0 images are representative of leaky lesions, while a representative non-leaky lesion is shown in the day 14 angiogenic group.

**Figure 3:**
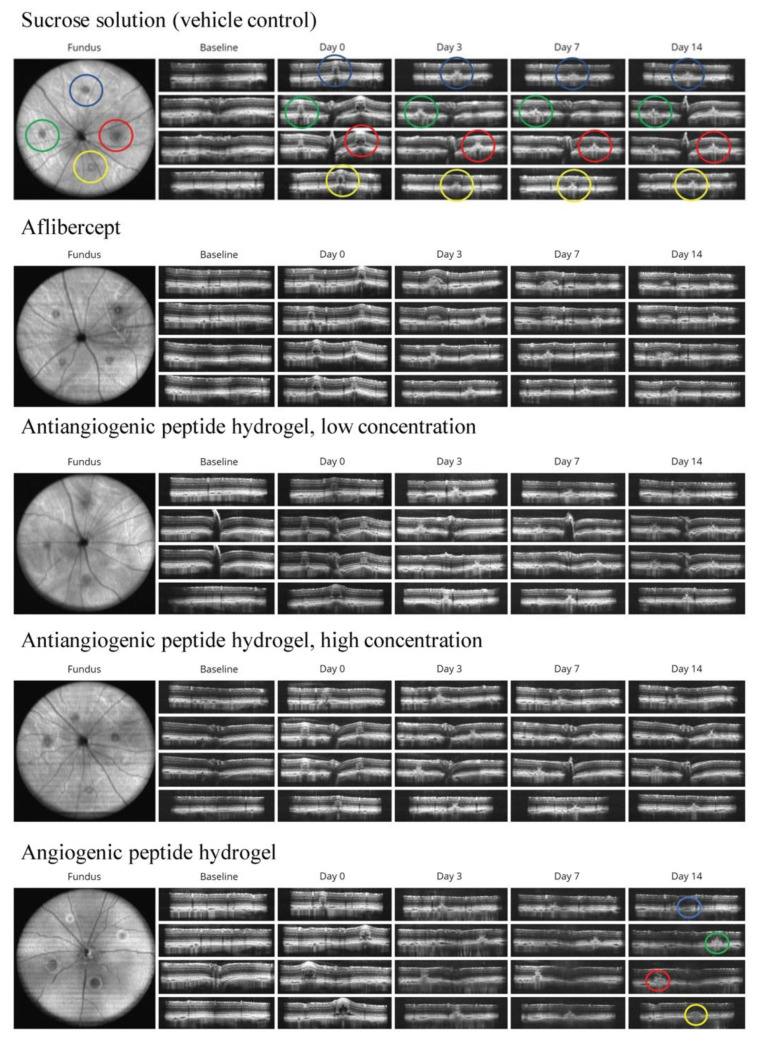
Representative SC-ODT images from experimental groups at baseline, immediately after photocoagulation on day 0, and follow ups on days 3, 7, and 14. The presence of intraretinal fluid in SD-OCT images suggests the presence of CNV; circles in the fundus image outline the location of each laser photocoagulation spot (approximately at 12 o’clock (blue), 3 o’clock (red), 6 o’clock (yellow) and 9 o’clock (green)) over different timepoints showing the development of CNV for each group. The four fundus images were taken through the retina containing one of the four lesions. Day 0 images are representative of leaky lesions, while a representative non-leaky lesion is shown in the day 14 angiogenic group.

The presence of leaky CNV lesions was determined from FA (Figure 2) and SD-OCT (Figure 3) images acquired immediately after CNV induction (day 0 in Figure 3), on day 3, day 7, and day 14 post-CNV.

**Figure 4:**
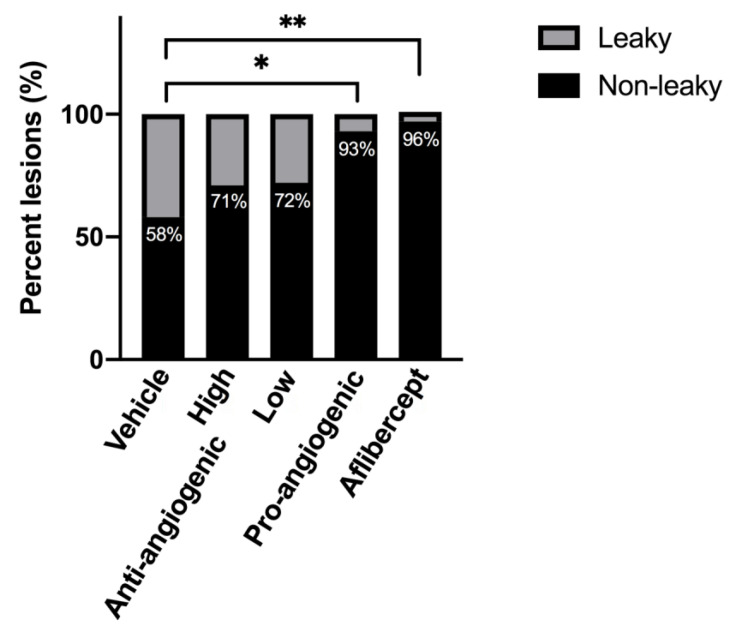
Number of lesions without leaky vessels at day 14 based on in vivo FA and SD-OCT imaging of experimental groups: sucrose vehicle, anti-angiogenic peptide hydrogel (high), anti-angiogenic peptide hydrogel (low), pro-angiogenic peptide hydrogel, and Aflibercept, respectively. The pro-angiogenic peptide hydrogel and Aflibercept showed protective effects compared to the vehicle group (* *p* < 0.05, ** *p* < 0.01).

We first analyzed CNV lesions qualitatively. Lesions were graded as “leaky” or not “leaky” by a researcher experienced in the CNV model (Figure 4). On day 7, only 4.2% of lesions treated with Aflibercept and 7.1% of lesions treated with pro-angiogenic peptide hydrogel had the presence of leaky lesions, significantly less than lesions treated with vehicle solution (41.7%, *p* = 0.002 and *p* = 0.003, respectively). The treatment effects of pro-angiogenic peptide hydrogel were comparable to that of Aflibercept (*p* = 0.65). Lesions treated with either low concentration or high concentration of anti-angiogenic peptide hydrogel showed statistically non-significant benefit compared to vehicle solution (low concentration 28.1% non-leaky *p* = 0.29 and high concentration 29.2% non-leaky *p* = 0.33, respectively). On day 14, all treatment groups showed a higher percentage of leaky CNV lesions, with a statistically significant difference compared to the vehicle group. Lesions treated with pro-angiogenic peptide hydrogel had the lowest percentage of leaky lesions (57.1%), followed by high concentration of anti-angiogenic hydrogel (62.5%), and low concentration of anti-angiogenic hydrogel (71.9%). Aflibercept treated lesions had a similar percentage of leaky lesions compared to the vehicle control group (75%).

### 3.3. Quantitative Analysis of CNV Lesions

**Figure 5:**
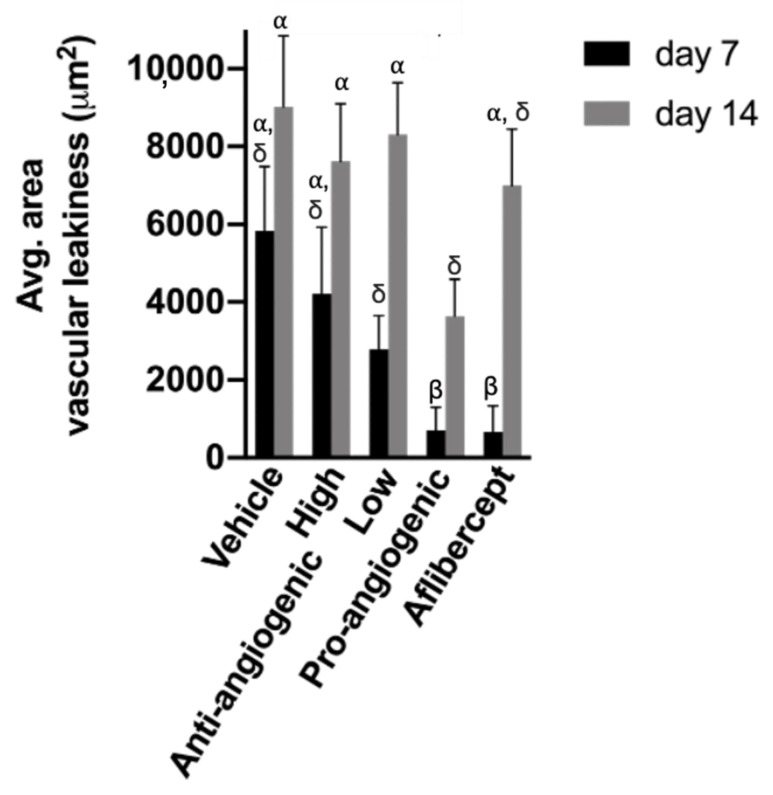
Average area of vascular leak at day 7 and day 14 based on fluorescein angiography imaging of experimental groups: vehicle, anti-angiogenic peptide hydrogel (high), anti-angiogenic peptide hydrogel (low), pro-angiogenic peptide hydrogel, and Aflibercept. At day 7, the pro-angiogenic peptide hydrogel and Aflibercept groups had smaller area of vascular leakiness compared to the vehicle group (*p* < 0.05). Similar Greek letter show no significant difference between groups.

CNV lesions were analyzed quantitatively by evaluating the average areas of vascular leak per lesion based on FA images (Figure 5). On day 0, average lesion areas were similar among all groups (*p* > 0.05). On day 7, the lesions treated with pro-angiogenic peptide hydrogel and Aflibercept had a significantly smaller area of vascular leak compared to lesions treated with vehicle solution (*p* = 0.007). The average area of vascular leak was similar in pro-angiogenic peptide hydrogel group and Aflibercept group (714.3 μm^2^ vs. 666.7 μm^2^, respectively, *p* = 0.96). The average area of vascular leak in the high and low concentrations of anti-angiogenic peptide hydrogel groups were 4208.3 μm^2^ and 2781.3 μm^2^, non-statistically significantly smaller than that in the vehicle group (5833.3 μm^2^, *p* = 0.50 and 0.11, respectively).

On day 14, all treatment groups showed higher average area of vascular leak, with no statistically significant difference compared to the vehicle group. The area of vascular leak was the lowest in the pro-angiogenic peptide hydrogel group (3642.9 μm^2^), and the highest in the vehicle group (9025.0 μm^2^).

## 4. Discussion

In this study, we demonstrated the tolerability and therapeutic efficacy of pro-angiogenic and anti-angiogenic peptide hydrogels in a choroidal neovascularization (CNV) rat model and demonstrated the potential for pro-angiogenic peptide hydrogels performing comparably to Aflibercept. In addition, pro-angiogenic and anti-angiogenic peptide hydrogels suggest potentially quicker effects than Aflibercept.

Treatment strategies for wet AMD have evolved in the last decade, drastically changing patient outcomes. Before the advent of anti-VEGF therapy for wet AMD, patients would progressively lose vision and become permanently blind. With the approval of Ranibizumab and Aflibercept for wet AMD and the off-label use of Bevacizumab, visual acuity after treatment initially improves or stabilizes. However, more than 70% of patients do not maintain driving vision in the long term [55]. These therapeutics also have other limitations, including the need to indefinitely receive intravitreal injections due to non-lasting effects and relatively short half-lives at 4–8 days. Some patients require injections as frequently as monthly. Each intravitreal injection carries the risk of endophthalmitis, retinal tear, ocular hypertension, retinal hemorrhage, and iatrogenic cataract, in addition to subjective risks such as anxiety and pain [56].

Our study yielded the surprising results that both the pro-angiogenic and anti-angiogenic peptide hydrogel showed significant improvement in CNV lesions comparable to Aflibercept. We hypothesized that the pro-angiogenic peptide hydrogel may stabilize vasculature and prevent the vascular permeability that results in leaky lesions. It has been observed that hypoxia contributes to the pathogenesis of vascular permeability and fragility in wet AMD. Pro-angiogenic peptide hydrogel may protect vascular integrity by improving oxygen delivery in neovasculature [57]. Additionally, it has been observed that different isoforms of VEGF have different effects on vascular permeability [58]. Therefore, it is possible that the pro-angiogenic peptide was structurally more like the VEGF isoforms that do not induce vascular permeability. Previous publications have shown that pro-angiogenic peptide hydrogel can promote maturation of new vessels [43,50], thereby preventing vascular permeability in immature vessels [59].

## 5. Conclusions

There are several limitations to our study. We used a small number of animals and follow up time was limited to 14 days, a short time frame for a chronic disease. The effect of pro-angiogenic and anti-angiogenic peptide hydrogels showed trends of improvement compared to vehicle control and Aflibercept, but the differences were not statistically significant due to the small number of animals studied.

The pro-angiogenic hydrogel showed significant treatment effects comparable to the standard of care Aflibercept in both the number of leaky lesions and the area of vascular leak. In addition, both pro-angiogenic and anti-angiogenic peptide hydrogels showed moderate improvement of CNV lesions at day 14. The pro-angiogenic peptide hydrogel showed more favorable treatment responses at day 14, suggesting the possibility of longer lasting effects than Aflibercept. Pro-angiogenic and anti-angiogenic self-assembling peptide hydrogels could potentially provide several advantages compared to existing anti-VEGF intravitreal injections.

In conclusion, the preclinical study suggests that pro-angiogenic peptide hydrogels are comparable to Aflibercept at treating CNV in rats. Follow up studies in larger animal preclinical studies will be needed to elucidate the total length of activity with pro-angiogenic and anti-angiogenic peptide hydrogels.

## Data Availability

The data presented here are available on request from the corresponding author.

## Supplementary Materials

The following are available online at https://www.mdpi.com/article/10.3390/bioengineering8120190/s1, Figure S1: Body weight changes between experimental groups, Figure S2: HPLC trace for pro-angiogenic peptide, Figure S3: HPLC trace for anti-angiogenic peptide.
