# Supplementary material for: Preclinical Efficacy of Pro- and Anti-Angiogenic Peptide Hydrogels to Treat Age-Related Macular Degeneration
Source: Bioengineering (Basel). 2021 Nov 23;8(12):190. doi: 10.3390/bioengineering8120190 (PMC8698576; doi:10.3390/bioengineering8120190)
Supplement: Supplementary file 1 [file bioengineering-08-00190-s001.zip › bioengineering-1407848-supplementary.pdf]

bioengineering

Supplementary Materials

The supplementary material contains HPLC traces for the pro- and anti-angiogenic peptides, as well as body weight changes between experimental groups.

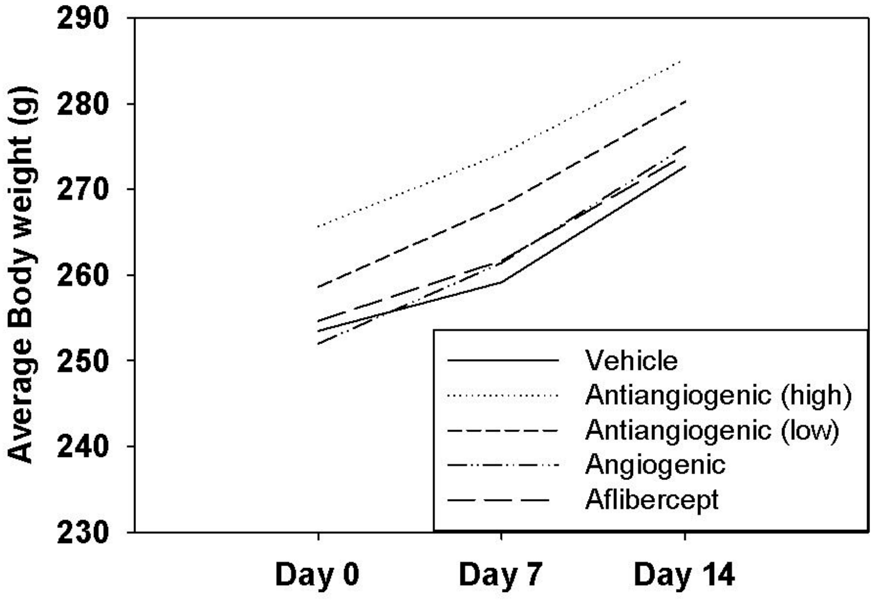

Supplemental Figure 1. Body weight changes between experimental groups. No effect of treatment (P = 0.542) on body weight among experimental groups: vehicle, anti-angiogenic peptide hydrogel (high), anti-angiogenic peptide hydrogel (low), pro-angiogenic peptide hydrogel, and Aflibercept. No significant effects of treatment on body weight were found through Two-Way Repeated Measures ANOVA comparison.

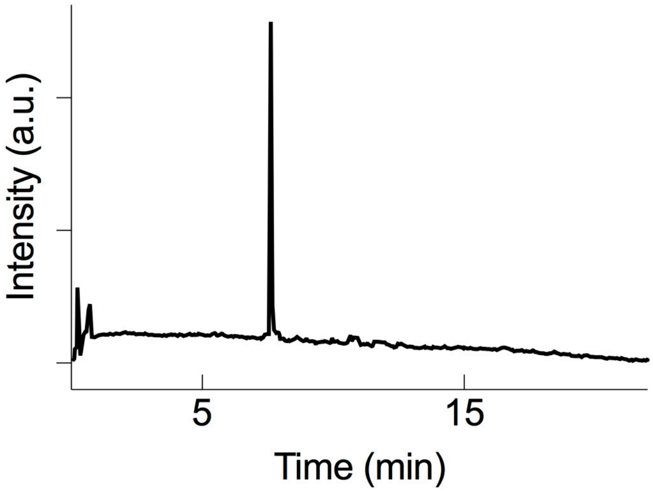

Supplemental Figure 2. High Performance Liquid Chromatography (HPLC) UV trace for pro-angiogenic peptide, monitored at 280 nm.

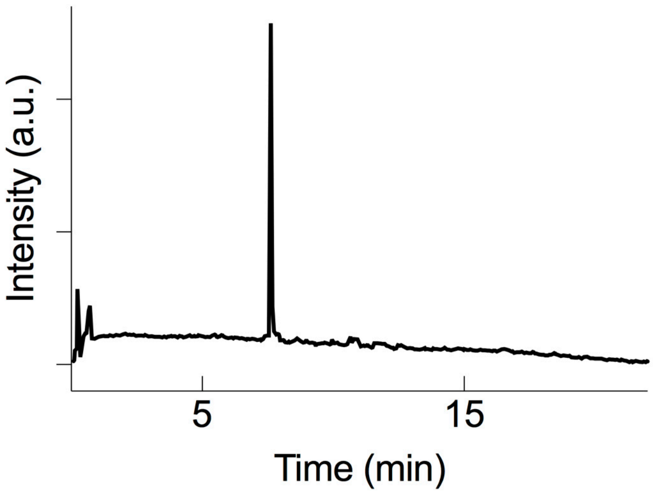

Supplemental Figure 3. High Performance Liquid Chromatography (HPLC) UV trace for anti-angiogenic peptide, monitored at 280 nm.
